# Immunoregulatory properties of erythroid nucleated cells induced from CD34+ progenitors from bone marrow

**DOI:** 10.1371/journal.pone.0287793

**Published:** 2023-06-30

**Authors:** Julia A. Shevchenko, Roman Yu Perik-Zavodskii, Kirill V. Nazarov, Vera V. Denisova, Olga Yu. Perik-Zavodskaya, Yulia G. Philippova, Alaa Alsalloum, Sergey V. Sennikov

**Affiliations:** Laboratory of Molecular Immunology, Federal State Budgetary Scientific Institution “Research Institute of Fundamental and Clinical Immunology”, Novosibirsk, Russia; The University of Texas MD Anderson Cancer Center, UNITED STATES

## Abstract

CD 71+ erythroid nucleated cells have pronounced immunoregulatory properties in normal and pathological conditions. Many populations of cells with immunoregulatory properties are considered candidates for cellular immunotherapy for various pathologies. This study characterized the immunoregulatory properties of CD71+ erythroid cells derived from CD34-positive bone marrow cells under the influence of growth factors that stimulate differentiation into erythroid cells. CD34-negative bone marrow cells were used to isolate CD71+ erythroid nuclear cells. The resulting cells were used to assess the phenotype, determine the mRNA spectrum of the genes responsible for the main pathways and processes of the immune response, and obtain culture supernatants for the analysis of immunoregulatory factors. It was found that CD71+ erythroid cells derived from CD34+ cells carry the main markers of erythroid cells, but differ markedly from natural bone marrow CD71+ erythroid cells. The main differences are in the presence of the CD45+ subpopulation, distribution of terminal differentiation stages, transcriptional profile, secretion of certain cytokines, and immunosuppressive activity. The properties of induced CD71+ erythroid cells are closer to the cells of extramedullary erythropoiesis foci than to natural bone marrow CD71+ erythroid cells. Thus, when cultivating CD71+ erythroid cells for clinical experimental studies, it is necessary to take into account their pronounced immunoregulatory activity.

## Introduction

Erythropoiesis is a tightly regulated multi-stage process that originates from a multipotent stem cell and ends with a mature enucleated erythrocyte [1]. Human erythropoiesis includes the stages of early erythropoiesis, terminal erythroid differentiation, and maturation of reticulocytes [2]. The terminal erythroid differentiation phase is subdivided chronologically into four stages, proerythroblast (ProE), basophilic erythroblast (BasoE), polychromatophilic erythroblast (PolyE), and orthochromatic erythroblast (OrthoE), based on the morphological characteristics of the cells [3]. The main function of erythroid nucleated cells is to maintain a pool of circulating erythrocytes for respiratory gas exchange, but these cells involve other functions, including interaction with the immune system [4].

Many populations of induced or immature cells with immunoregulatory properties are considered as candidates for cellular immunotherapy in various pathological conditions: for example, regulatory T-cells [5], myeloid suppressor cells [6], mesenchymal stromal cells [7]. CD71+ erythroid cells, as immature cells, are usually absent or very rare in the blood of healthy adults, but are abundant in the spleen of newborn mice and in human umbilical cord blood and have immunoregulatory properties [8]. CD71+ erythroid cells provide feto-maternal tolerance; are responsible for the susceptibility of newborns to infections [9–11]; infiltrate the tumor and contribute to its progression [12]; accumulate in the peripheral blood during SARS-CoV-2 infection, leading to hypoxia, anemia, and coagulopathy in moderate to severe infection [13, 14]; reduce the production of pro-inflammatory cytokines by myeloid cells and reduce the activation and secretion of cytokines by lymphocytes and promote the production of anti-inflammatory cytokines and the differentiation of naive CD4+ T-cells into various types of T-cells, T-helper cells type 2 (Th2) [15], induced T-regulatory cells [8]. An analysis of the heterogeneity of erythroid precursors from the yolk sac, fetal liver, cord blood, and bone marrow in humans has shown that a population of immunoerythroid cells with dual erythroid and immunoregulatory networks is present at all stages of human ontogenesis. It is the immunomodulatory subgroup, and not the entire population of erythroid precursors, that may represent the cellular basis of immunoregulatory functions [16] However, the potential contribution of erythroid nucleated cells to non-respiratory physiological processes still requires detailed study. Therefore, studies aimed at further characterizing the immunological role of erythroid nucleated cells are of great importance. Since we assume that erythroid cells with pronounced immunoregulatory properties can be used for nonspecific immunotherapy, we conducted a study of the immunoregulatory properties of erythroblasts obtained in vitro from CD34-positive (CD34+) progenitor cells from the bone marrow.

## Materials and methods

### Donors

Bone marrow sampling from healthy adult donors was approved by the local ethics committee of the Research Institute of Fundamental and Clinical Immunology at meeting No. 129 on 17 February 2021. Each donor signed an approved informed consent form and underwent a general clinical examination (a general clinical blood test and electrocardiogram; a negative test for COVID-19, measurement of pressure and pulse immediately before the bone marrow sampling procedure). The age of the healthy bone marrow donors was 27.33 ± 6.34 years (mean ± standard error of the mean), and the study population consisted of three males and three females. All healthy donors for the study were recruited between February and December 2021. Bone marrow was obtained by puncture of the ilium. Mononuclear cells were isolated by the standard method via a Ficoll–Urografin gradient (Ficoll: PanEco, Moscow, Russia; Urografin: Schering AG, Germany). For this purpose, the bone marrow was diluted with Dulbecco’s solution (Biolot, St. Petersburg, Russia) at a ratio of 1:1, layered on the Ficoll–Urografin mixture (PanEco, Moscow, Russia) at a Ficoll/bone marrow ratio of 1:3, and centrifuged for 25 min at 312 RCF (Elmi, Latvia). Cells of the interphase ring were collected and washed twice with Dulbecco’s solution.

### Cell culture

CD34+ cells were isolated from the obtained mononuclear cells through positive magnetic selection by means of the CD34 MicroBead kit Ultra Pure Kit (MicroBeads conjugated to mouse monoclonal anti-human CD34 antibodies, isotype mouse IgG1, Cat# 130-046-702, Miltenyi Biotec, Germany); the CD34+ cells were next used to prepare induced CD71+ erythroid cells as described before [17]. Namely, CD34+ cells (10 000/ml) were cultured for 7 days (expansion stage) in the X-VIVO 15 serum-free medium (Lonza, Switzerland) supplemented with 25 ng/ml rhSCF (Biolegend, San Diego, United States), 50 ng/ml rhTPO (Biolegend, San Diego, United States), 50 ng/ml Flt3 ligand (Gibco, Thermo Fisher Scientific, USA), and 10 μl/ml of the 100x sodium selenite-insulin-transferrin (ITS) growth supplement (Biolot, St. Petersburg, Russia).

Cell differentiation was implemented in the next stage of cell cultivation. To this end, the cells (2 × 10^5^/ml) were cultivated for 3 days with supplementation of 3 IU/ml erythropoietin (the State Research Institute of Highly Pure Biopreparations, affiliated with the Federal Medico-Biological Agency, St. Petersburg, Russia), 25 ng/mL rhSCF (Biolegend, San Diego, United States), 10 ng/mL rhIL-3 (Biolegend, San Diego, United States), and 10 ng/ml rhIL-6 (Biolegend, San Diego, United States). The third stage (cell maturation) involved culturing 1*10^4^ cells/ml in the new portion of the medium X-VIVO supplemented with erythropoietin. The maturation stage continued for the next 5 days. After that, we took the supernatant for cytokine production analysis, and the cells were used for gene expression analysis and phenotyping. Cells were washed twice after each stage of culturing. We placed the cells in a fresh portion of the culture medium at each subsequent stage of cultivation. We collected the cell supernatant to determine the content of cytokines after the third stage of cultivation. During this period, cells were cultured in a medium supplemented with erythropoietin, which is absent in the Bio-Plex Pro Human Cytokine 48-Plex Screening Panel (Bio-Rad Laboratories, USA), so there was no background effect of cytokines that we added to differentiate erythroid cells.

CD34-negative (CD34–) bone marrow mononuclear cells were used to isolate CD71+ erythroid nucleated cells by positive magnetic selection for marker CD71 (MicroBeads conjugated to mouse monoclonal anti-human CD71 antibodies, clone: AC108.1, isotype mouse IgG2a, Cat# 130-046-201, Miltenyi Biotec, Germany). Next, we took 2*10^5^ cells after magnetic sorting for RNA isolation. The other part of the CD71+ erythroid cellscells was at a concentration of 1*10^6^ cells/ml and cultured for 72 hours in X-VIVO 15 medium supplemented with 10 μl/ml of 100x insulin transferrin. The culture supernatant was collected for analysis of immunoregulatory factors. and for a mixed lymphocyte reaction (see below) as a conditioned medium.

### Flow cytometry

By this procedure, the resultant cells were analyzed for markers CD45 Brilliant Violet 510 (mouse monoclonal antibodies against human antigen CD45, clone HI30, Isotype Mouse IgG1, κ, Cat# 304036, Biolegend, San Diego, United States), CD71-PE (mouse monoclonal antibodies against human antigen CD71, clone CY1G4, Isotype Mouse IgG2a, κ, Cat# 334106, Biolegend, San Diego, United States), and CD235a-FITC (mouse monoclonal antibodies against human antigen CD235a (Glycophorin A), clone HI264, Isotype Mouse IgG2a, κ, Cat# 349104, Biolegend, San Diego, United States). Antibodies to markers CD45-PerCP/Cyanine5.5 (mouse monoclonal antibodies against human antigen CD45, clone HI30, Isotype Mouse IgG1, κ, Cat# 304028, Biolegend, San Diego, United States), CD3-FITC (mouse monoclonal antibodies against human antigen CD3, clone OKT3, Isotype Mouse IgG2a, κ, Cat# 317306, Biolegend, San Diego, United States), CD4-PE/Cyanine7 (mouse monoclonal antibodies against human antigen CD4, clone RPA-T4, Isotype Mouse IgG1, κ, Cat# 300512, Biolegend, San Diego, United States), CD8a-APC/Cyanine7 anti-human (mouse monoclonal antibodies against human antigen CD8, clone HIT8a, Isotype Mouse IgG1, κ, Cat# 300926, Biolegend, San Diego, United States), CD16-PE (mouse monoclonal antibodies against human antigen CD16, clone B73.1, Isotype Mouse IgG1, κ, Cat# 360704, Biolegend, San Diego, United States), and CD19-APC (mouse monoclonal antibodies against human antigen CD19, clone HIB19, Isotype Mouse IgG1, κ, Cat# 302212, Biolegend, San Diego, United States) were used to detect lymphoid-cell markers. For staining by flow cytometry, all antibodies, except for CD 235a, were used at a dose of 5 μl per 1 million cells in 100 μl of cell suspension. For the CD 235a antibody, we used 0.5 μg per 1 million cells in 100 μl of cell suspension. These doses of antibodies were recommended by the manufacturer. The analysis was performed on an Attune NxT Flow Cytometer (Thermo Fisher Scientific, USA).

### Gene expression profiling by means of the nanostring nCounter GX human immunology v2 kit

Total RNA was isolated from a 200,000-cell sample with the Total RNA Purification Plus Kit (Norgen Biotek, Canada). Concentration and quality of RNA in each sample were assessed on a Nanodrop 2000 spectrophotometer (Thermo Fisher Scientific, USA). All the RNA samples were diluted to 10 ng/μl with nuclease-free water, and the diluted total RNA was frozen at –80°C until analysis. Gene expression profiling with the help of the Nanostring nCounter SPRINT Profiler analytical system was performed on 50 ng of total RNA from each sample. The RNA samples were analyzed by means of the nCounter Human Immunology v2 panel, which consists of 579 immune and inflammation-associated genes, 15 housekeeping genes, and eight negative and six positive controls; these controls were utilized to subtract background signals and to normalize the numbers of unprocessed mRNA transcripts in all samples. The samples were subjected to an overnight hybridization reaction at 65°C, where 5–12 μl of total RNA was combined with 3 μl of nCounter Reporter probes, 0–7 μl of DEPC-treated water, 10 μl of hybridization buffer and with 5 μl of nCounter capture probes (total reaction volume was 30 μl). After the hybridization of the probes to targets of interest in the samples, the number of target molecules was determined on the nCounter digital analyzer and evaluated on the nSolver platform. The gene expression profiles were examined in the nSolver 4. nSolver 4 was used to perform QC and to perform normalization using added synthetic positive controls and the 15 housekeeping genes included in the panel. Then background thresholding was performed on the normalized data. The background thresholding procedure returned 50 genes with above noise level expression. The noise level was determined as: mean of negative controls + 2 standard deviations + 40

We performed differential gene expression using multiple T-tests (with Q < 0.05) in GraphPad Prism 9.4. The Volcano- and MA-plots were created in GraphPad Prism 9.4. The GSEA was done using GSEApy (https://github.com/zqfang/GSEApy). The heatmap was created using bioinfokit (https://github.com/reneshbedre/bioinfokit).

### Cytokine secretion analysis

Assessment of cytokine secretion (concentrations) in culture supernatants of CD71+ erythroid cells was performed using the Bio-Plex Pro Human Cytokine 48-Plex Screening Panel (Bio-Rad Laboratories, USA). This assay platform is based on fluorescent-bead technology combined with a sandwich immunoassay, allowing for stand-alone and multiplex assays of up to 100 analytes in a single microtiter plate well. For this assay, a panel of 47 cytokines was employed in 96-well microtiter plate format. Contents of each well were analyzed using the Bio-Plex 100 System (Bio-Rad Laboratories) to quantify each specific reaction on the basis of the color of the particles and fluorescent-signal intensity. The data were finally processed in the Bio-Plex Manager software (version 6.1) by five-parameter curve fitting and were converted to picograms per milliliter.

### Mixed lymphocyte reaction

Peripheral blood was drawn from donors into vacuum tubes containing EDTA as an anticoagulant (Improvacuter, China). Peripheral blood mononuclear cells (PBMCs) were isolated from whole blood samples using a conventional Ficoll–Urografin density gradient method. Briefly, peripheral blood was diluted with an equal volume of RPMI-1640 medium (Biolot, St. Petersburg, Russia), then layered on a Ficoll-Urografin solution (ρ = 1.077 g/L) and centrifuged at 400× g and room temperature for 40 min. Mononuclear cells were collected from an opalescent layer located at the phase boundary over the entire tube cross-section. The RPMI-1640 medium supplemented with 10% fetal calf serum (FCS) (Biowest, Nuaillé, France), 2 mM L-glutamine (Biolot, Saint Petersburg,Russia), 5 × 10−4 M 2-mercaptoethanol (Sigma-Aldrich, St. Louis, MO, USA), 25 mM HEPES (Biolot, Saint Petersburg, Russia), 80 μg/mL gentamicin (KRKA, Novomesto, Slovenia) and 100 μg/mL benzylpenicillin (Biolot, Saint Petersburg, Russia) (hereinafter designated the culture medium) was used for PBMCs cultivation. “Effector” cells were dispensed into wells at 10^5^ cells/well. To block proliferative activity of “inducer” cells, they were treated with mitomycin C (Sigma, Germany) at 50 μg/ml for 60 min, washed three times with the culture medium, and were added in the amount of 10^5^ cells/well to the “effectors.” The cells were incubated in 200 μl of the culture medium at 37°C, 5% CO2, and 100% humidity for 4 days. This assay was carried out in 96-well round-bottom microtiter plates (TPP, Switzerland), in three identical wells with similar samples analyzed in parallel. The conditioned medium (culture supernatant) from the studied CD71+ erythroid cells constituted half of the total volume of a well during the cultivation. The proliferation rate of the cultured cells was assessed by means of the WST reagent (Takara Bio, Japan), which was added at 4 h before the end of cultivation. The results are presented as optical density.

### Statistic

Statistical analysis of the data to find differences between groups was conducted in the GraphPad Prism 8.4 and 9.4 software. Cytokine production was evaluated with the CytokineExplore online tool (http://exabx.com/apps/cytokineexplore/). The Mann–Whitney test was performed to compare the production of cytokines between the different types of CD71+ erythroid cells. ANOVA with multiple comparison criteria was carried out to evaluate statistical significance of the differences between the cell groups. Results were assumed to be statistically significant at P-value < 0.05, and the data are presented as a median with an interquartile range.

## Results

### Phenotypic characteristics of the induced CD71+ erythroid cells

**Fig 1:**
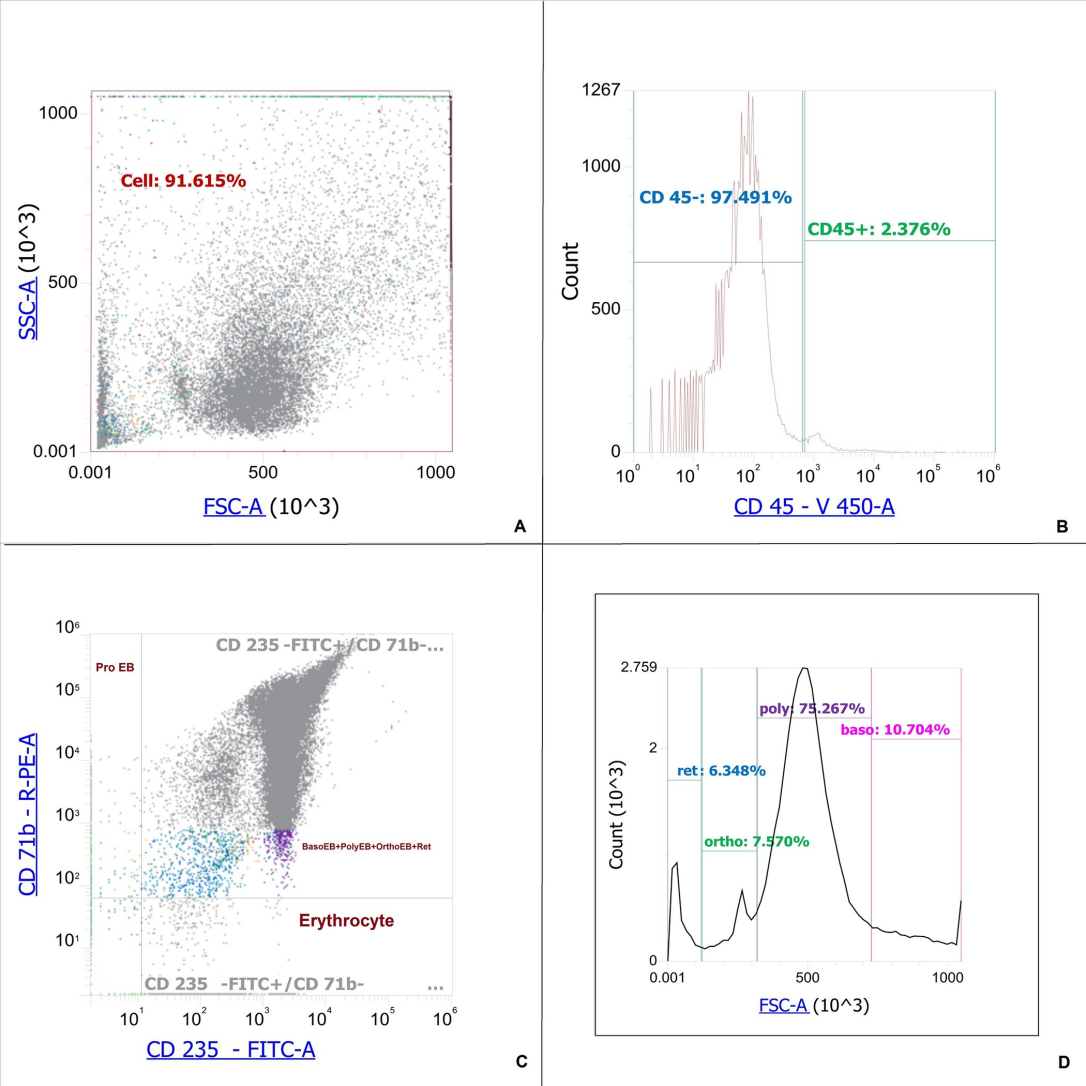
Staging of natural bone marrow CD71+ erythroid cells. A–Dot-plot for natural bone marrow CD71+ erythroid cells. B–Histogram of the distribution of bone marrow CD71+ erythroid cells by marker CD 45. C–Dot-plot of the distribution of bone marrow CD71+ erythroid cells by marker CD 71 and CD 235a from CD 45-negative natural bone marrow CD71+ erythroid cells, ProEB–proerythroblasts (CD235a^low^CD71^high^ cells), BasoEB+PolyEB+OrthoEB+Ret–basophilic erythroblasts, polychromatophilic erythroblasts, orthochromatophilic erythroblasts/reticulocytes (CD235^high^CD 71^pos^ cells), Erythrocytes–CD235a^high^CD71^neg^. D–Histogram of the distribution of stages of development of erythroid cells with a phenotype CD235^high^CD 71^pos^ depending on the FSC. Ret–reticulocytes (CD 235a^high^CD71^pos^ FSC^low^), Ortho–orthochromatophilic erythroblasts (CD235a^high^CD71^pos^FSC^low^ cells), Poly–polychromatophilic erythroblasts (CD235a^high^CD 71^pos^FSC^middle^ cells), Baso–basophilic erythroblasts (CD 235a^high^ CD71^pos^ FSC^high^ cells).

**Fig 2:**
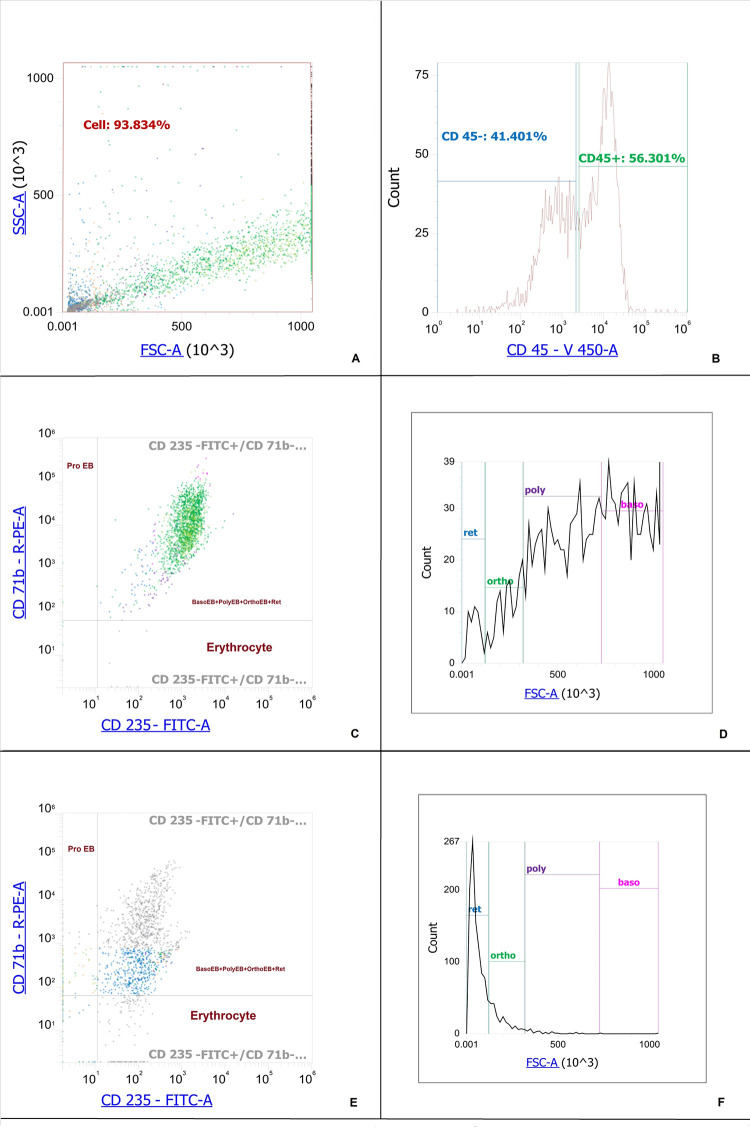
Staging of induced CD71+ erythroid cells derived from CD34+ bone marrow cells. A–Dot-plot for induced CD71+ erythroid cells derived from CD34+ bone marrow cells. B–Histogram of the distribution of induced CD71+ erythroid cells derived from CD34+ bone marrow cells by marker CD 45. C–Dot-plot of the distribution of bone marrow CD71+ erythroid cells by marker CD 71 and CD 235a from CD 45-positive induced CD71+ erythroid cells. D–Histogram of the distribution of stages of development of CD71+ erythroid cells with a phenotype CD45^pos^CD235a^high^CD 71^pos^ depending on the FSC. E–Dot-plot of the distribution of bone marrow CD71+ erythroid by marker CD 71 and CD 235a from CD 45-negative induced CD71+ erythroid cells. F–Histogram of the distribution of stages of development of CD71+ erythroid cells with a phenotype CD45^neg^CD235a^high^CD 71^pos^ depending on the FSC, ProEB–proerythroblasts (CD235a^low^CD71^high^ cells), BasoEB+PolyEB+OrthoEB+Ret–basophilic erythroblasts, polychromatophilic erythroblasts, orthochromatophilic erythroblasts/reticulocytes (CD235a^high^CD 71^pos^ cells), Erythrocytes–CD235a^high^CD71^neg^, Ret–reticulocytes (CD 235a^high^CD71^pos^ FSC^low^), Ortho–orthochromatophilic erythroblasts (CD235a^high^CD71^pos^FSC^low^ cells), Poly–polychromatophilic erythroblasts (CD235a^high^CD 71^pos^FSC^middle^ cells), Baso–basophilic erythroblasts (CD 235^high^ CD71^pos^ FSC^high^ cells).

CD71+ erythroid cells are a heterogeneous population with several stages of differentiation. The cells of each stage realize their unique functions, which are difficult to separate at the level of the whole organism. We determined the phenotype and maturation stage of CD71+ erythroid cells using the expression of CD 71, CD 235a and FSC [18, 19]. According to these sources, we differentiated proerythroblasts as CD235a^low^CD71^high^ cells, basophilic erythroblasts as CD 235^high^ CD71^pos^ FSC^high^ cells, polychromatophilic erythroblasts as CD235a^high^CD 71^pos^FSC^middle^, orthochromatophilic erythroblasts/reticulocytes as CD235^high^CD71^pos^FSC^low^, erythrocytes CD235a^high^CD71^neg^. The population of orthochromatophilic erythroblasts/reticulocytes CD 235^high^CD71^pos^ FSC^low^ was clearly divided into two parts by FSC, so we were able to differentiate orthochromatophilic erythroblasts and reticulocytes from each other. It was found that induced erythroblasts can be divided into a CD45+ population and a CD45– population (Table 1). The distribution of natural bone marrow CD71+ erythroid cells and induced CD71+ erythroid cells is shown in Figs 1 and 2, respectively.

**Table 1:** Proportions (%) of CD45+ and CD45- cells among the induced CD71+ erythroid cells derived from CD34+ bone marrow cells and bone marrow CD71+ erythroid cells. The data are presented as the mean ± standard derivation (n = 6).

	Bone marrow erythroblasts	Induced erythroblasts
CD45+ cells	2.47±0.6	46.74±9.28
CD45– cells	97.53±0.6	52.59±9.31

**Fig 3:**
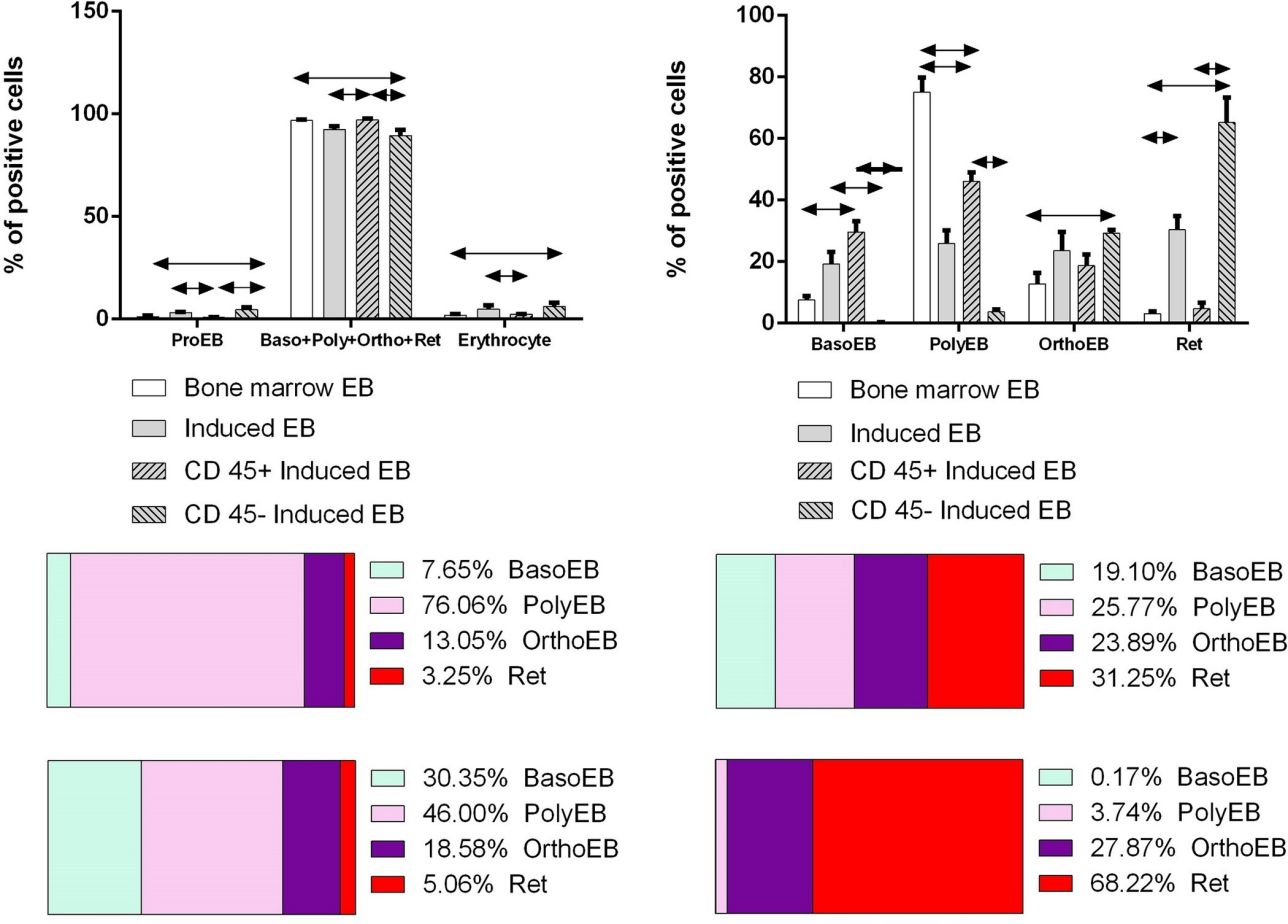
Comparison of the distribution of stages of development of bone marrow CD71+ erythroid cells and CD71+ erythroid cells induced from CD34+ bone marrow cells. Kruskal-Wallis test ANOVA with Dunn’s correction for multiple comparisons was carried out to evaluate statistical significance of the differences between the cell groups. Results were assumed to be statistically significant at p-value < 0.05, and the data are presented as a median with an interquartile range.

We compared the content of CD71+ erythroid cells of different stages of development for natural and induced CD71+ erythroid cells (Fig 3).

Our data show that in the bone marrow of a healthy adult, polychromatophilic erythroblasts predominate. The general population of induced erythroblasts differs from bone marrow cells by a decrease in the content of polychromatophilic erythroblasts and the redistribution of all other stages towards the accumulation of more mature forms. At the same time, basophilic and polychromatophilic erythroblasts predominate among CD 45-positive CD71+ erythroid cells, while orthochromatophilic and reticulocytes predominate among CD 45-negative ones. Thus, induced CD71+ erythroid cells retain their phenotypic identity with bone marrow CD71+ erythroid cells, but their distribution by stages differs markedly.

**Fig 4:**
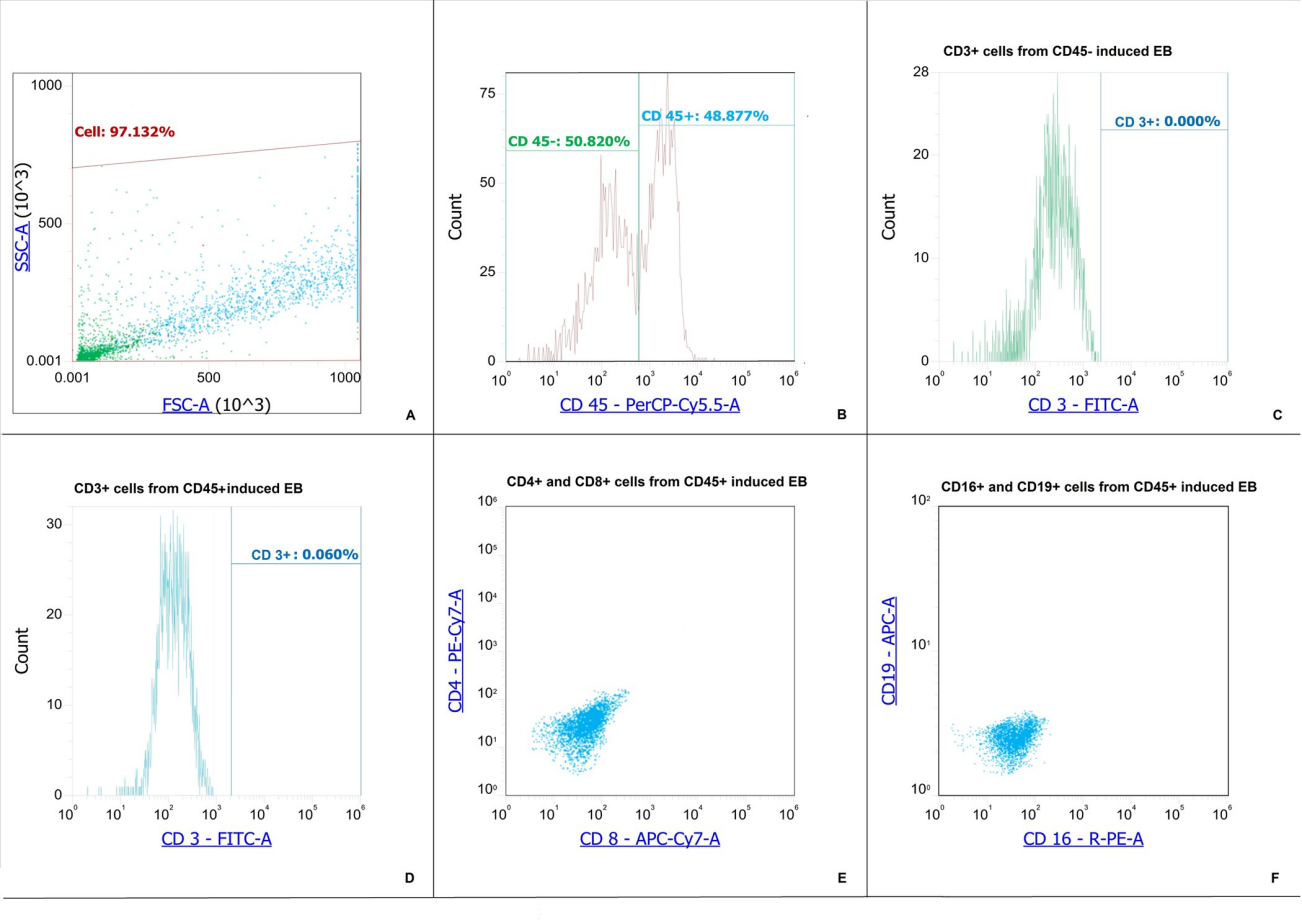
Expression of lymphoid cell markers on the surface of induced CD71+ erythroid cells. A–Dot-plot for induced CD71+ erythroid cells derived from CD34+ bone marrow cells. B–Histogram of the distribution of induced CD71+ erythroid cells derived from CD34+ bone marrow cells by marker CD 45. С –Histogram of the distribution of induced CD71+ erythroid cells derived from CD34+ bone marrow cells by marker CD 3 from CD 45-negative induced CD71+ erythroid cells. D–Histogram of the distribution of induced CD71+ erythroid cells derived from CD34+ bone marrow cells by marker CD 3 from CD 45-positive induced CD71+ erythroid cells. E–Dot-plot of the distribution of bone marrow CD71+ erythroid cells by marker CD 8 and CD 4 from CD 45-positive induced erythroblasts. F–Dot-plot of the distribution of bone marrow CD71+ erythroid cells by marker CD 16 and CD 19 from CD 45-positive induced erythroblasts.

To determine the feasibility of deriving a lymphoid lineage from the CD34+ cells, we quantified the expression of markers CD3, CD4, CD8, CD16, and CD19. Induced CD71+ erythroid cells from bone marrow CD 34+ cells had the phenotype CD45+CD3–CD4–CD8–CD16–CD19– and CD45–CD3–CD4–CD8–CD16–CD19– (Fig 4).

### Gene expression analysis

**Fig 5:**
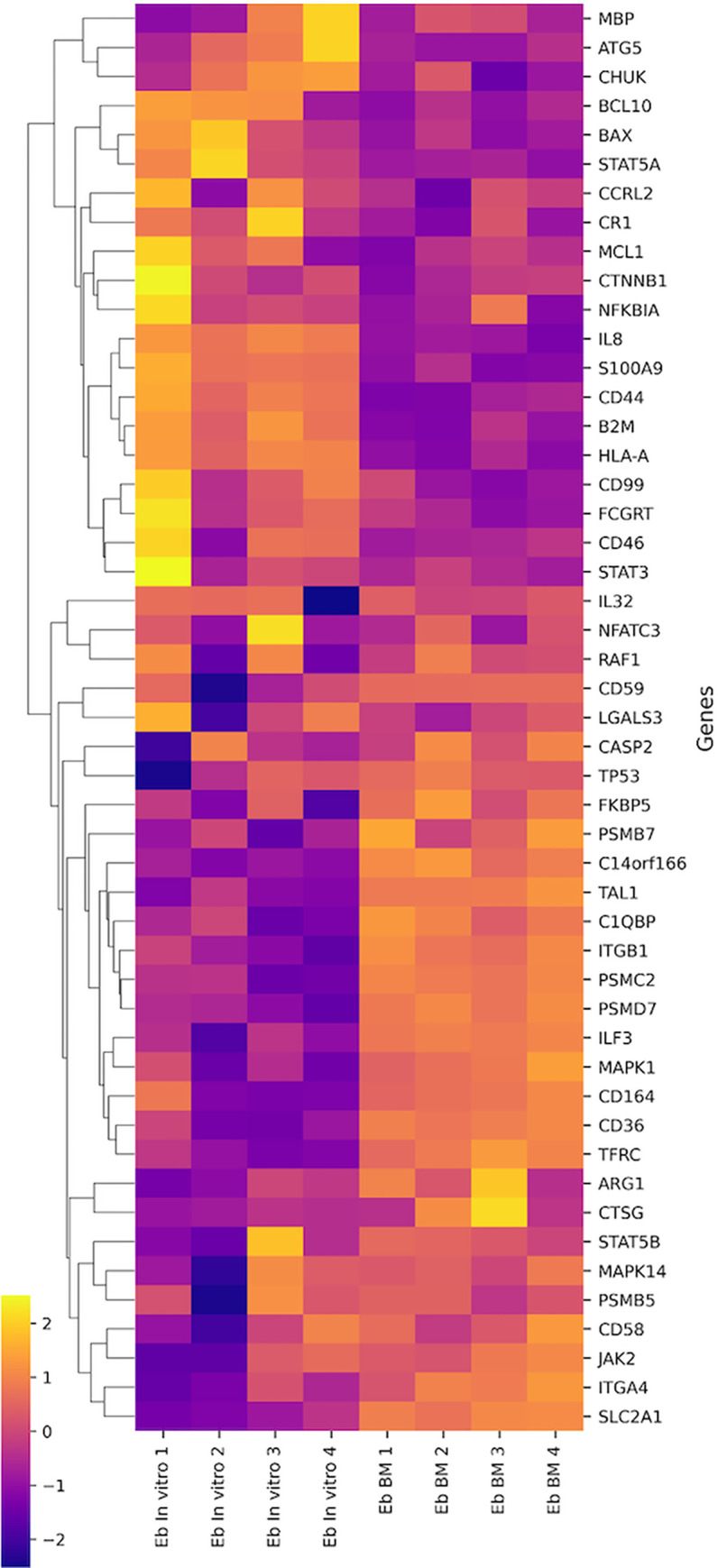
Heat map of studied cells’ transcriptome.

We examined the expression of 579 genes associated with inflammation and immune response. Analysis of the expression profile of natural and induced erythroblasts revealed differential expression of 49 genes (Fig 5).

Most differentially expressed genes are related to pathogen interaction processes, innate immune system, cytokine signaling, adaptive immune system, lymphocyte activation, hemostasis, apoptosis, T- and B-cell signaling. The minimum numbers of differentially expressed genes are related to autophagy processes, Th17-, Th1-, Treg-differentiation, signaling through TGF-b and Type I Interferon.

**Fig 6:**
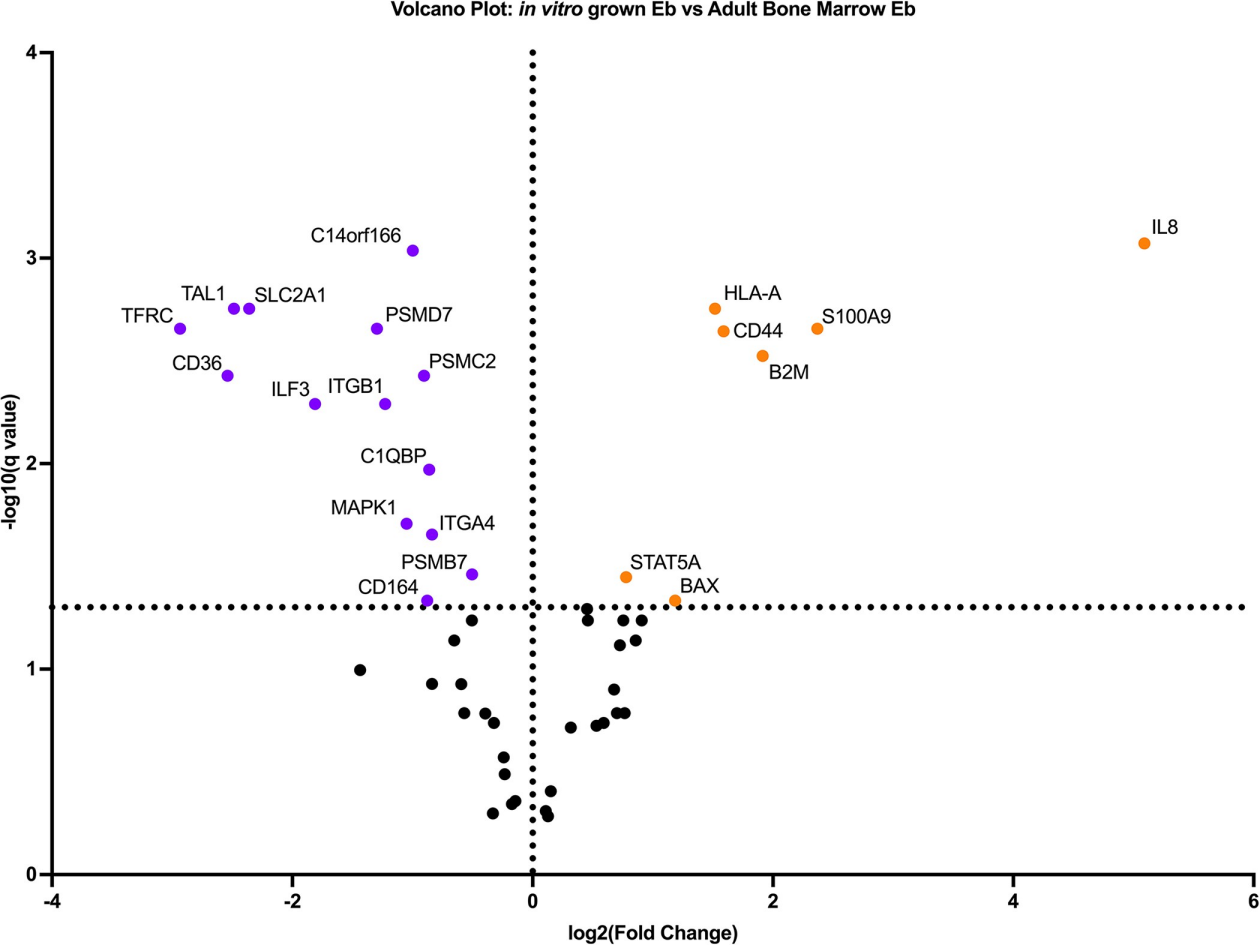
Volcano-plots of differentially expressed genes. The purple dots correspond to the samples of induced CD71+ erythroid cells, the orange dots correspond to the samples of natural CD71+ erythroid cells of bone marrow.

**Fig 7:**
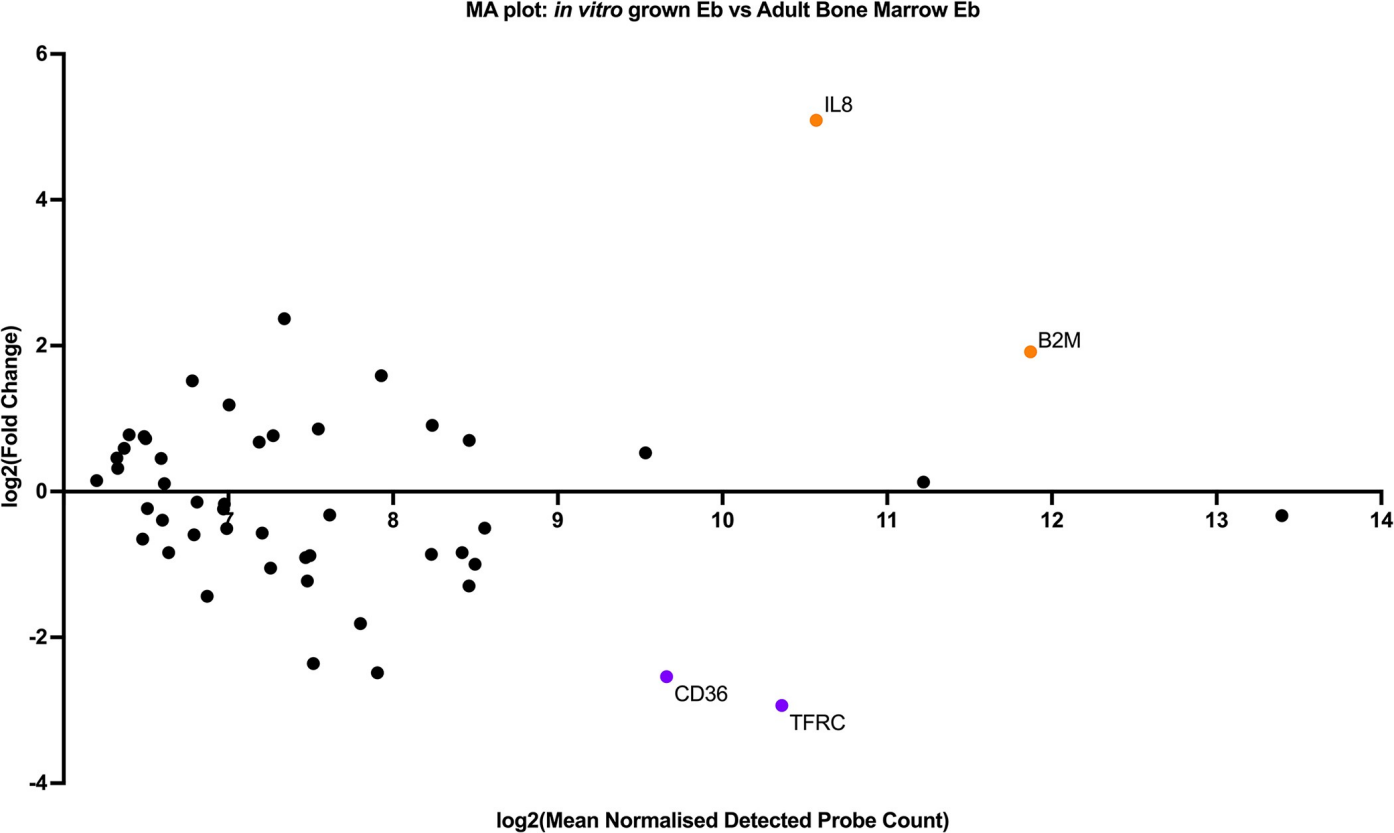
MA-plots of differentially expressed genes. The purple dots correspond to the samples of induced CD71+ erythroid cells, the orange dots correspond to the samples of natural CD71+ erythroid cells of bone marrow.

As studied cells had obvious transcriptomic heterogenecity we performed differential gene expression analysis. To do this, we built Volcano- and MA- plots of differentially expressed genes A volcano plot (Fig 6) is a type of scatterplot that shows statistical significance (P-value) versus magnitude of change (fold change). It enables quick visual identification of genes with large fold changes that are also statistically significant. These may be the most biologically significant genes. In a volcano plot, the most upregulated genes are towards the right, the most downregulated genes are towards the left, and the most statistically significant genes are towards the top. MA-plot (Fig 7) is a 2-dimensional (2D) scatter plot used for visualizing gene expression datasets MA plot visualize and identify gene expression changes from two different conditions in terms of log fold change on Y-axis and log of the mean of normalized expression counts of two conditions on X-axis. Generally, genes with lower mean expression values will have highly variable log fold changes. Genes with similar expression values will cluster around 0 value i.e genes expressed with no significant differences in between sampes. Points away from 0 line indicate genes with significant expression, For example, a gene is upregulated and downregulated if the point is above and below 0 line respectively.

The induced CD71+ erythroid cells are characterized by the predominance of the expression of B2M, CD44, HLA-A, IL-8 genes, which are involved in the processes of lymphocyte activation, cell adhesion, chemokine signaling, NLR signaling, type II Interferon Signaling, type I Interferon Signaling. CD71+ erythroid cells of the bone marrow are characterized by the predominance of expression of the ILF3, TAL1, and CD36 genes, which are involved in the regulation of transcription and oxidative stress.

**Fig 8:**
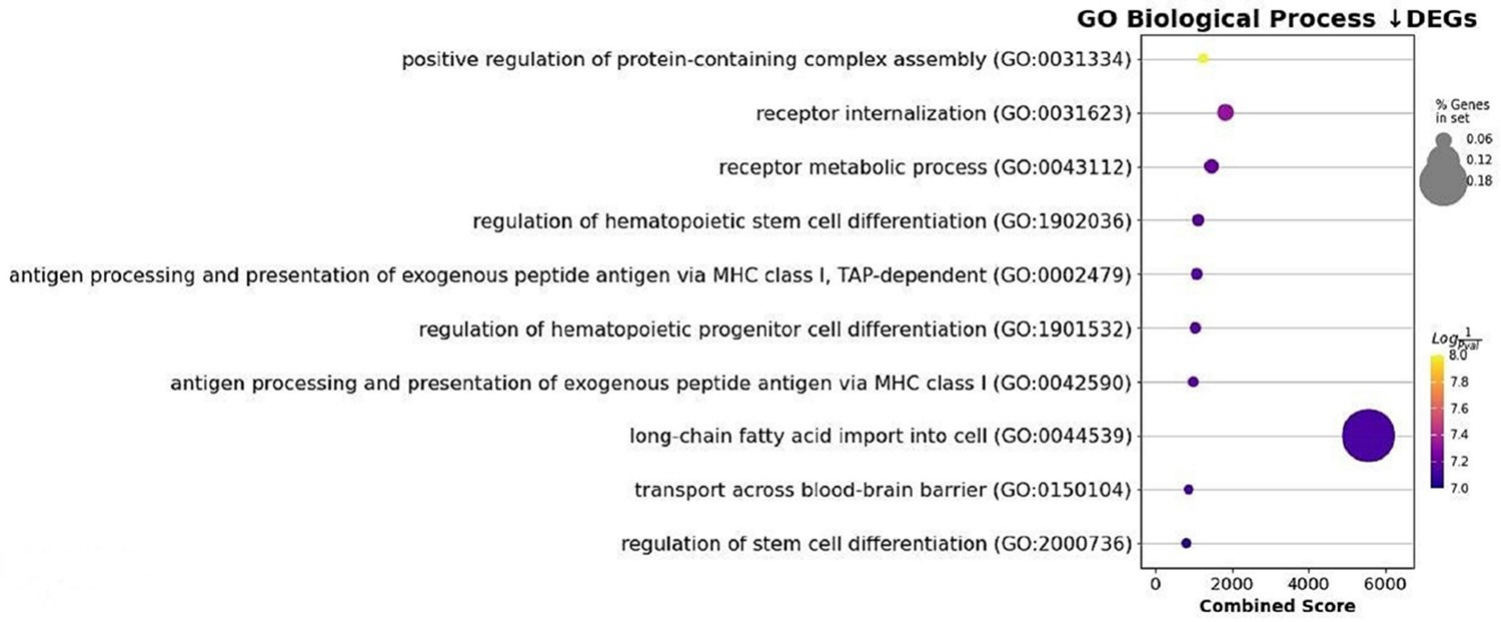
Plot of Gene Set Enrichment Analysis (GSEA) results showing down-regulated differentially expressed genes.

Analysis of the MA plot showed that CD71+ induced erythroid cells grown in vitro show significantly lower expression of early erythroblast-associated markers CD36, ITGA4 and TFRC and significantly higher expression of the IL-8 chemokine.To characterize the potential functions altered by DEG, we performed an enrichment analysis of GO pathways.using the “Gene Ontology Biological Process” terms (Fig 8). Notable terms were the “regulation of hematopoietic stem cell differentiation” and the “regulation of hematopoietic progenitor cell differentiation”.

### Analysis of cytokine production by natural and induced CD71+ erythroid cells

**Fig 9:**
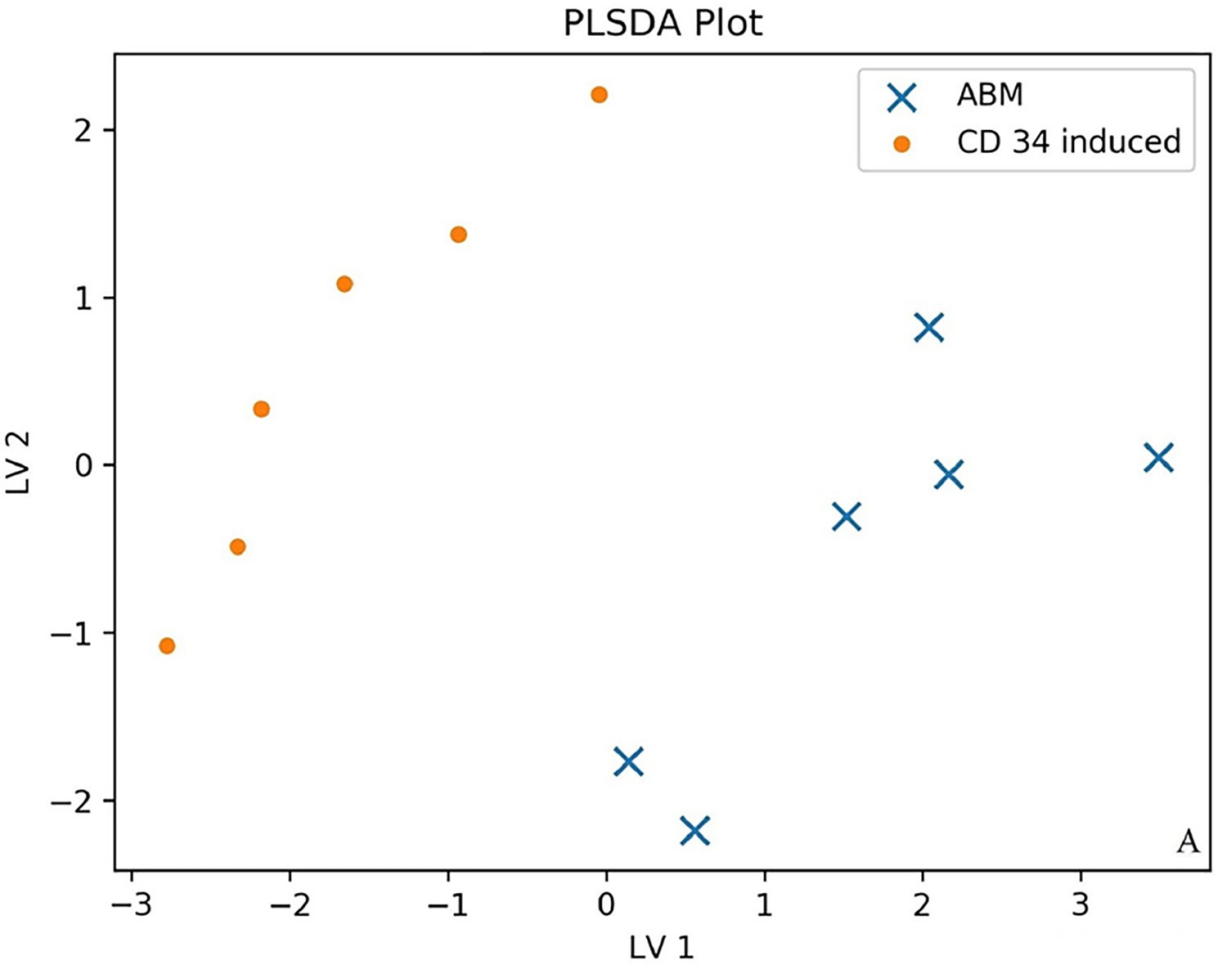
The PLS-DA plot of cytokine production by different types of CD71+ erythroid cells (n = 6 for bone marrow CD71+ erythroid cells and induced CD71+ erythroid cells from CD34^+^ bone marrow cells). An example distribution of data points for chemokines, interleukins, and growth factors is shown. (x)–ABM–bone marrow erythroblasts. (·)–CD 34 induced–induced erythroblasts from CD34^+^ bone marrow cells.

**Fig 10:**
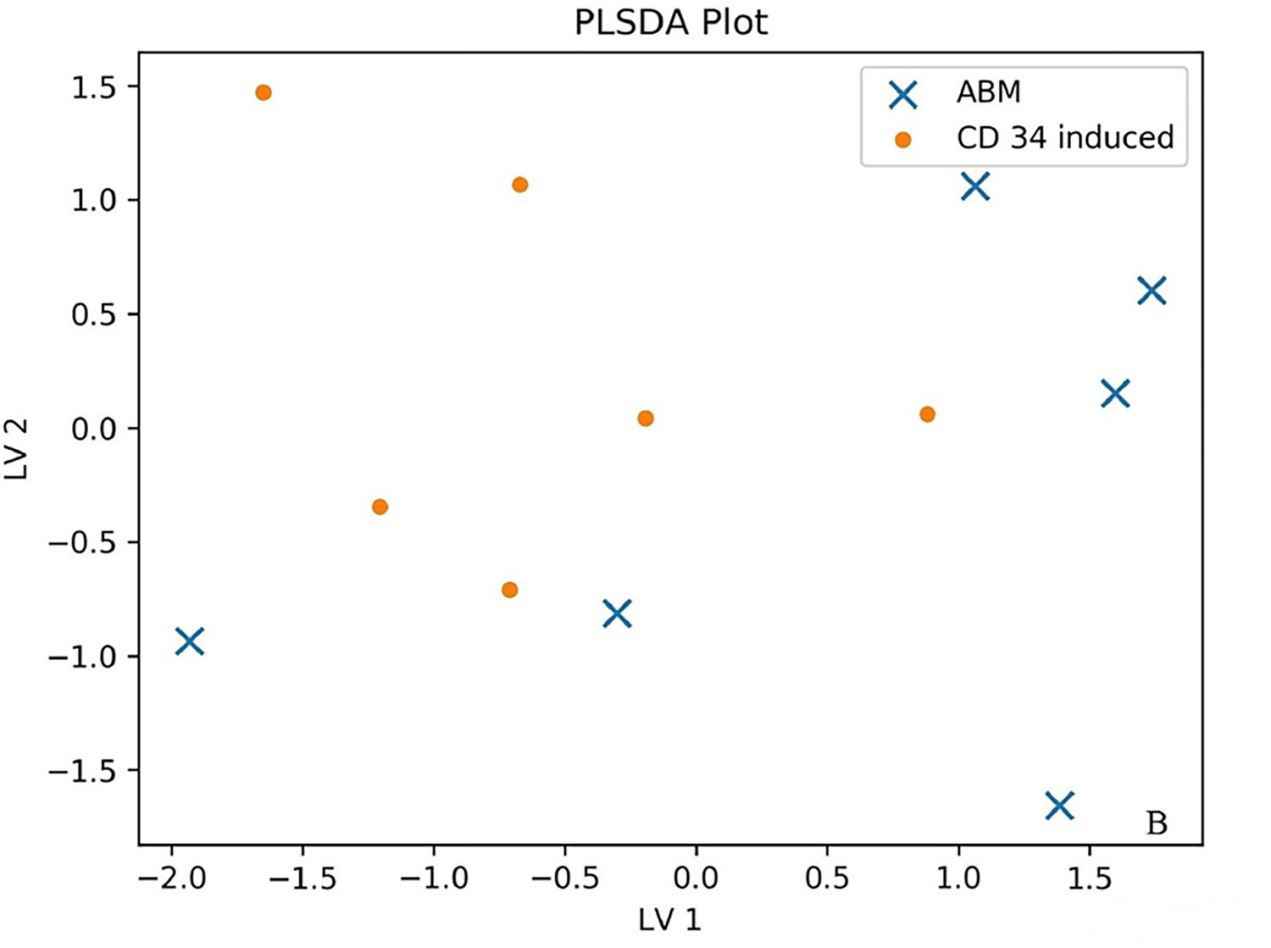
The PLS-DA plot of cytokine production by different types of CD71+ erythroid cells (n = 6 for bone marrow CD71+ erythroid cells and induced CD71+ erythroid cells from CD34^+^ bone marrow cells). An example distribution of data points for colony-stimulating factors, interferons, and TNF family cytokines is shown. (x)–ABM–bone marrow erythroblasts. (·)–CD 34 induced–induced erythroblasts from CD34^+^ bone marrow cells.

We investigated the production of 47 cytokines in conditioned media of natural bone marrow CD71+ erythroid cells and induced CD71+ erythroid cells. Partial least squares discriminant analysis (PLS-DA) and an importance plot of variables for two groups [20] were employed to identify differences in the production of cytokines between the two groups of CD71+ erythroid cells under study. Partial Least Squares Discriminant Analysis (PLSDA) makes a graph showing the projection multi-cytokine data on only two axes/components. The two groups are colored and symbolized differently. PLSDA is used when using partial least squares regression to problems with categorical outcomes (in our case, induced versus natural erythroblasts). Axles are marked LV1 (latent variable 1) and LV2 (latent variable 2). If the data points of the two groups overlap strongly, then this implies that the two groups have a similar cytokine profile. On the other hand, if the data points barely overlap, then this would mean that groups have very different profiles. As a precaution, if the number of samples is very small, and the number cytokines is high, the PLSDA plot may exaggerate the difference between groups. To this end, we subdivided all cytokines into functional groups: chemokines (10 cytokines: CTACK, Eotaxin, GRO-alpha, IP-10, MCP-1, MCP-3, MIF, MIG, MIP-1alpha, RANTES), interleukins (21 cytokines: IL-1alpha, IL-1betta, IL-1RA, IL-2, IL-2Ralpha, IL-3, IL-4, IL-5, IL-6, IL-7, IL-8, IL-9, IL-10, IL-12 (p70), IL-12 (p40), IL-13, IL-15, IL-16, IL-17, IL-18, LIF), growth factors (8 cytokines: basic FGF, HGF, betta-NGF, PDGF-BB, SCF, SCGF-betta, SDF-1alpha, VEGF), colony-stimulating factors (3 cytokines: G-CSF, GM-CSF, M-CSF), interferons (2 cytokines: IFN-alpha2, IFN-gamma) and TNF family cytokines (3 cytokines: TNF-alpha, TNF-betta, TRAIL) to reduce the number of false positive results in the context of the small number of samples and the large number of analytes. Data points of the production of chemokines, interleukins, and growth factors did not overlap between the two populations of CD71+ erythroid cells (natural and induced), indicating that the two cell groups have different cytokine production profiles (Fig 9). Data points of the production of colony-stimulating factors, interferons, and TNF family cytokines overlapped between the populations of the natural and induced CD71+ erythroid cells, suggesting that these cell groups have similar cytokine production profiles (Fig 10).

**Fig 11:**
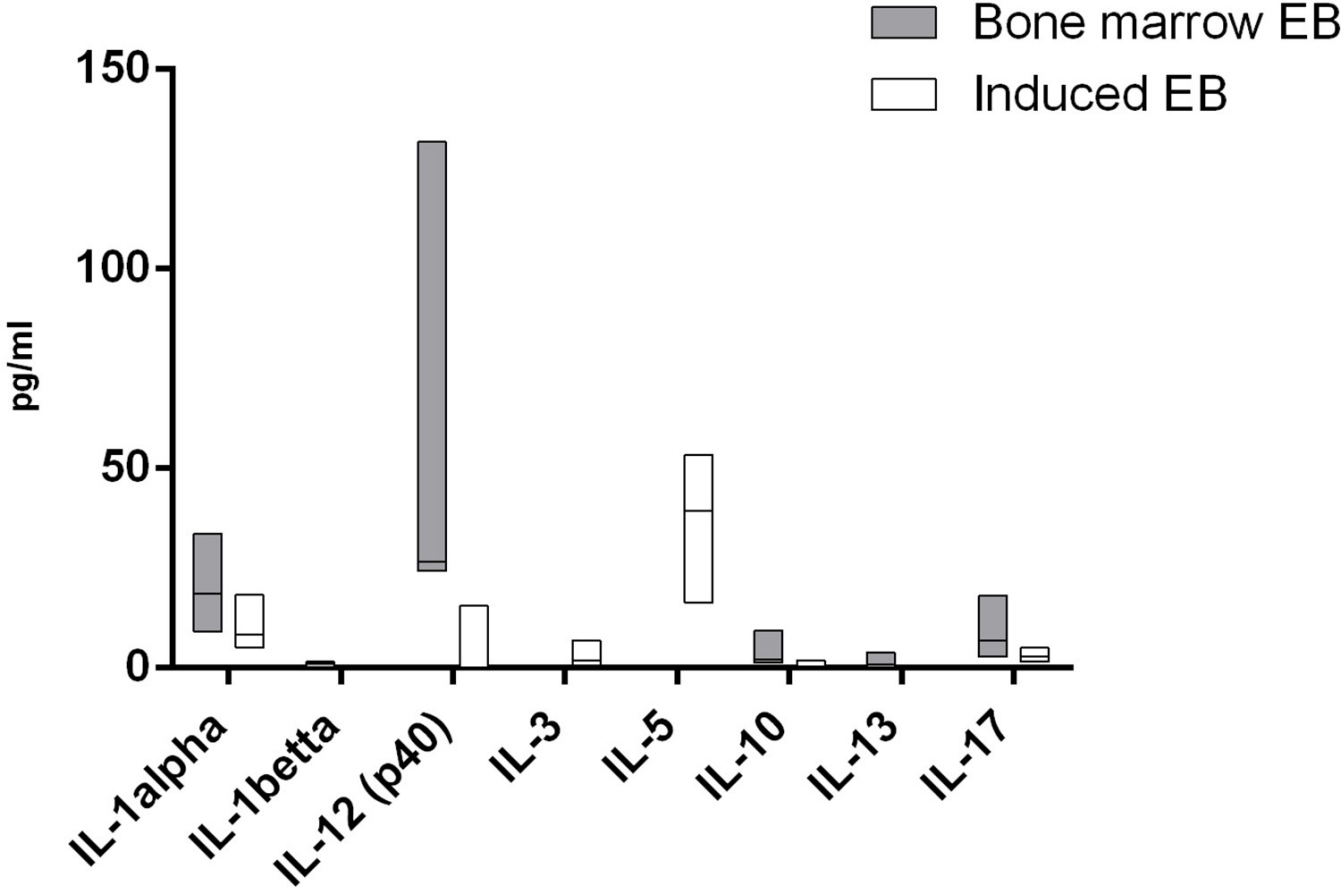
Cytokines that manifested significant differences in secretion of interleukins between the two types of CD71+ erythroid cells (n = 6 for bone marrow CD71+ erythroid cells and induced CD71+ erythroid cells from CD34^+^ bone marrow cells). The Mann–Whitney test was performed to compare the production of cytokines between the different types of CD71+ erythroid cells. Results were assumed to be statistically significant at *p*-value < 0.05, and the data are presented as a median with an interquartile range.

**Fig 12:**
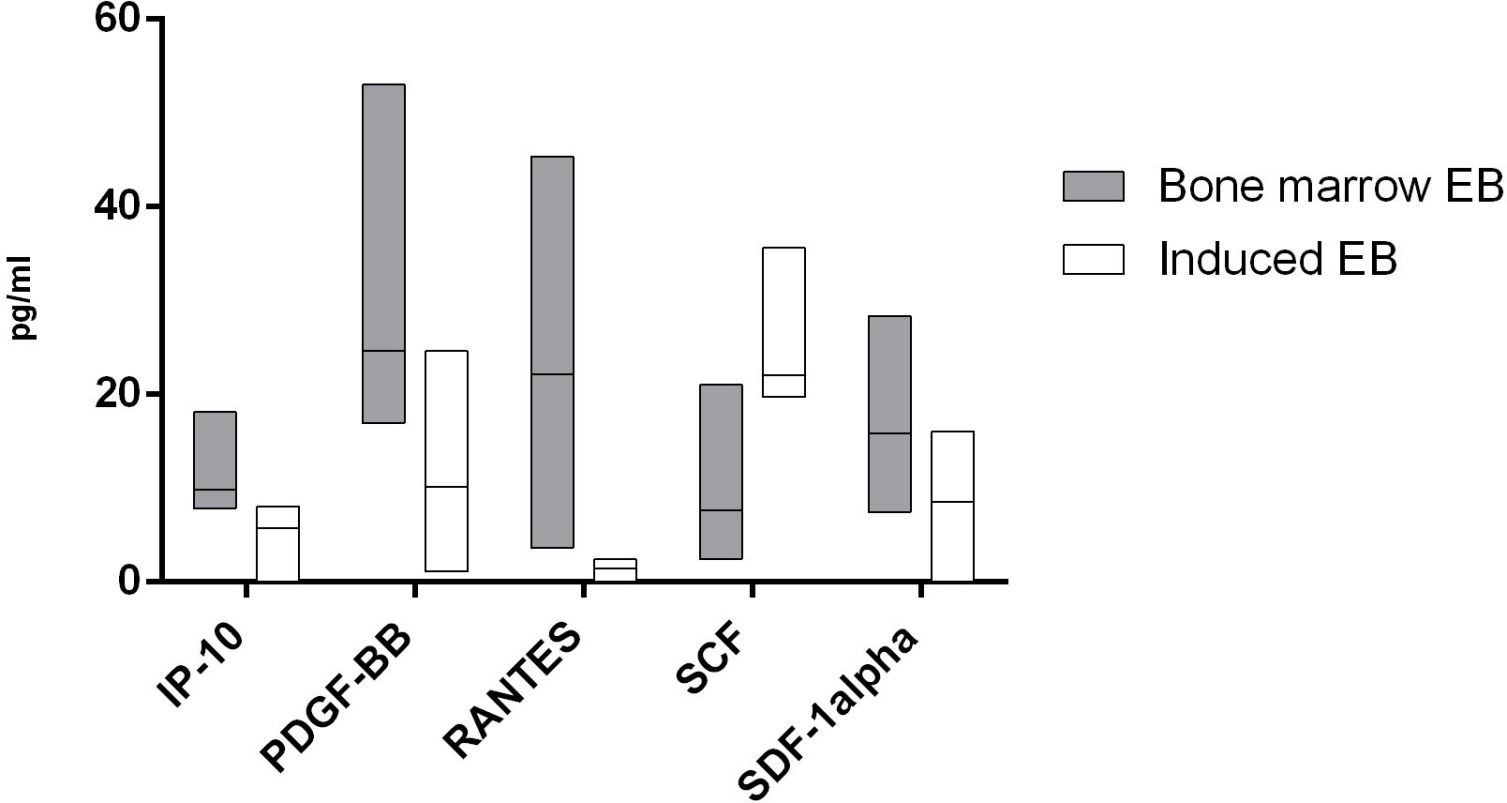
Cytokines that manifested significant differences in secretion of chemokines and growth factors between the two types of CD71+ erythroid cells (n = 6 for bone marrow CD71+ erythroid cells and induced CD71+ erythroid cells from CD34^+^ bone marrow cells). The Mann–Whitney test was performed to compare the production of cytokines between the different types of CD71+ erythroid cells. Results were assumed to be statistically significant at *p*-value < 0.05, and the data are presented as a median with an interquartile range.

For 13 cytokines, we found significant differences in the production of cells by cells of both studied groups. In a comparison of the cytokine production between the two types of erythroblasts, it was demonstrated that the natural CD71+ erythroid cells from bone marrow secrete significantly more IL-1β, IL-13, RANTES, IL-12 (p40), IL-1α, IL-10, IL-17, IP-10, PDGF-BB, and SDF-1α, whereas the induced CD71+ erythroid cells secrete larger amounts of IL-3, SCF, and IL-5 (Figs 11 and 12).

### Effect of the environment of CD71+ erythroid cells on the mixed culture of lymphocytes

To assess functional properties of the mediators secreted by the two types of CD71+ erythroid cells, the mixed lymphocyte reaction was carried out next. The principle behind this assay is as follows: T-cells from one donor will proliferate in the presence of antigen-presenting cells from another donor. The reason is the recognition of an HLA mismatch between two unrelated donors, which triggers an immune response of T cells. Mixed cultivation of lymphocytes is often used as a way to induce nonspecific stimulation/activation of T cells in culture [21]. To test the effects on antigen-presenting and allostimulatory functions of T cells, we used conditioned media from our natural erythroblasts and induced erythroblasts.

**Fig 13:**
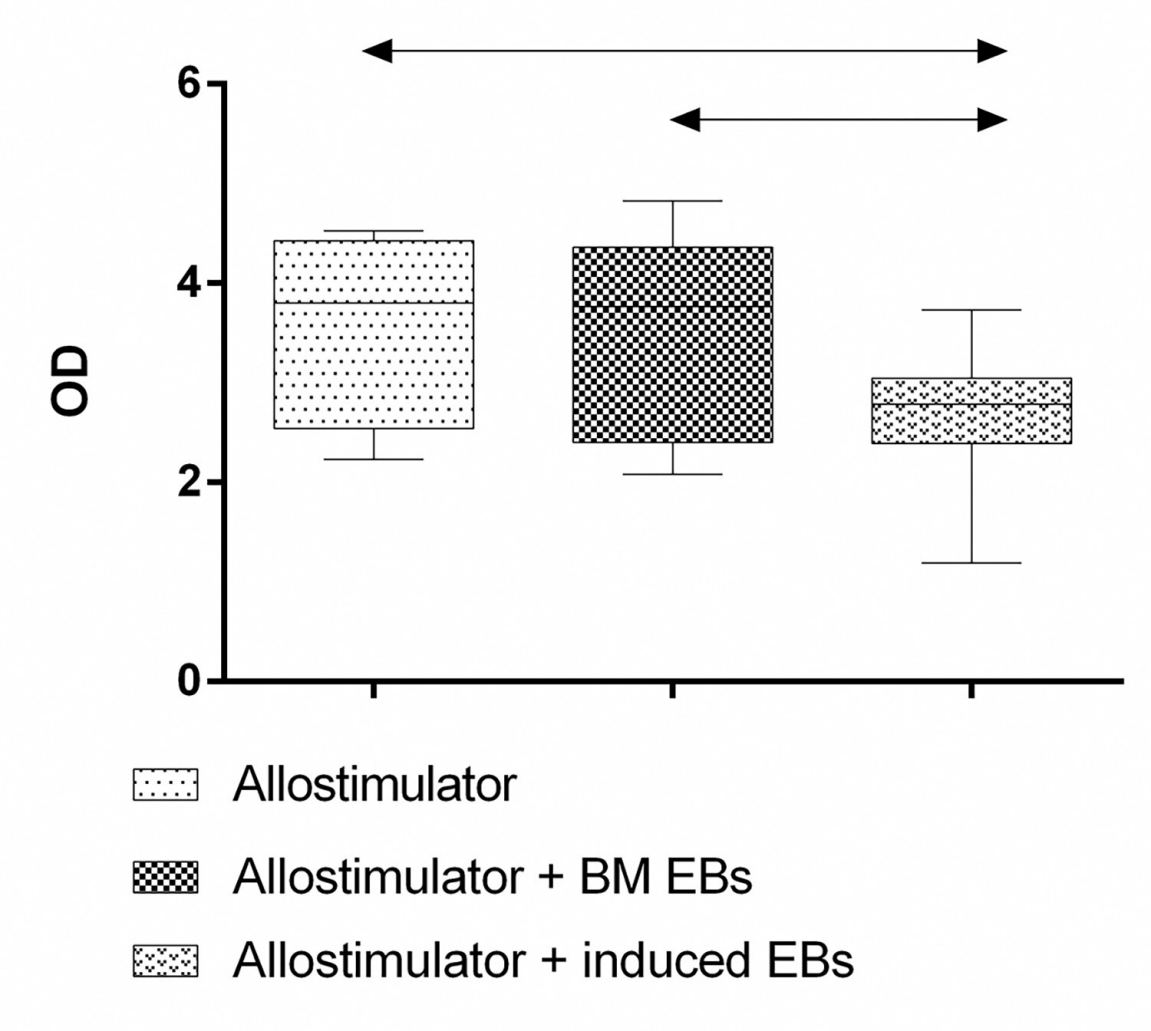
Effects of the conditioned media from either the natural or induced bone marrow CD71+ erythroid cells on the mixed lymphocyte reaction (n = 4). The results are presented as optical density of allogeneic cultures. Statistical analysis of the data was conducted in the GraphPad Prism 8.4 software. Two-way ANOVA with Tukey’s correction for multiple comparisons was carried out to evaluate statistical significance of the differences between the cell groups. Results were assumed to be statistically significant at *p*-value < 0.05, and the data are presented as a median with an interquartile range. “Allostimulator”: culture of mononuclear cells with the addition of allogeneic donor cells treated with mitomycin C; “Allostimulator + BM EBs”: culture of mononuclear cells with the addition of mitomycin C–treated allogeneic donor cells and supplementation with the conditioned medium from natural bone marrow CD71+ erythroid cells; “Allostimulator + induced EBs”: culture of mononuclear cells with the addition of mitomycin C–treated allogeneic donor cells and supplementation with the conditioned medium from the induced CD71+ erythroid cells derived from CD34+ bone marrow cells of healthy donors.

In the experiment on the influence of the conditioned medium from the natural CD71+ erythroid cells, it was demonstrated that proliferative activity stayed at the same level as when the cells were stimulated with the alloantigen, i.e. processes of presentation, recognition, and response to the alloantigen remained intact (Fig 13). On the contrary, when the conditioned medium from the induced CD71+ erythroid cells was applied, the proliferative activity proved to be suppressed in the mixed lymphocyte culture (Fig 13).

## Discussion

In vitro cultivation of CD34+ bone marrow cells during incubation with erythropoietin (EPO), SCF, and IL-3 [22] makes it possible to obtain erythroblasts for further research or for the creation of possible immunotherapeutic technologies. Induced CD71+ erythroid cells derived from in vitro CD34+ precursors consist of two populations: CD45+CD71+CD235aa+ cells and CD45–CD71+CD235a+ cells. CD 45+ CD71+ erythroid cells do not express lymphoid cell markers and are present throughout the entire erythroblast culture. Such CD45+ CD71+ erythroid cells are found in foci of extramedullary erythropoiesis in the spleen, where they suppress anti-infective and anti-tumor immunity in neonates and adults with malignancy. CD45+CD71+ erythroid cells suppress T cells due to the secretion of reactive oxygen species, IL-10 and TGF-β via the paracrine pathway and during intercellular contact, and their suppressor effect is more pronounced than that of suppressor cells of myeloid origin [23]. CD71+ erythroid cells expressing the CD45 panleukocyte marker at the earliest stages of differentiation are more powerful immunoregulators than CD45+ CD71+ erythroid cells at later stages [23, 24]. Currently, the presence of immunosuppressive properties of CD71+ erythroid cells with CD 45 expression is confirmed by their extramedullary presence in the bloodstream of patients with SARS-CoV-2 [25]. CD45+CD71+ erythroid cells downregulate effector CD8+ T cell activity (eg, granzyme B expression and degranulation [CD107] capacity) via reactive oxygen species [26]. Our study confirmed that the conditioned medium of induced CD71+ erythroid cells has a more pronounced inhibitory effect on the allogeneic mixed lymphocytic reaction compared to the conditioned medium from natural bone marrow CD71+ erythroid cells. This conclusion can be explained by the report that human bone marrow contains much fewer early-stage CD71+ erythroid cells than late-stage CD71+ erythroid cells [27]. In the present analysis of gene expression in the NanoString nCounter Sprint system, a difference in gene expression was shown between natural bone marrow CD71+ erythroid cells and cultured (induced) CD71+ erythroid cells. These data indicate that: CD71+ erythroid cells cultured *in vitro* were more mature than erythroblasts of bone marrow origin, since they have a significantly lower expression of the ITGA4, TFRC, and CD36 genes [28] program of homeostatic regulation of stationary erythropoiesis. The balance between production and destruction of red blood cells is tightly regulated to maintain the optimal concentration of red blood cells in the blood. Too few erythrocytes are not able to provide sufficient oxygen delivery to the tissues, and too many erythrocytes lead to an increase in blood viscosity, impaired blood flow, which also reduces oxygen delivery. Induced erythroblasts do not have restrictive regulatory requirements, which allows them to quickly pass through the differentiation program [29]. Analysis of gene expression showed that natural and induced CD71+ erythroid cells differ significantly in the processes of activation of the mechanisms of the innate and adaptive immune systems, activation of T- and B-cell signaling, activation and apoptosis of lymphocytes, but have a similar effect on the functional differentiation of lymphocytes.

Previously, it was shown that bone marrow erythroid cells are able to produce a wide range of cytokines that can regulate various stages of hemo- and immunopoiesis [30–35]. In addition, the same work revealed differences in the production of some cytokines between natural bone marrow erythroblasts and cells obtained de novo from erythroid colonies [36]. In the current study, multiplex analysis showed that both types of CD71+ erythroid cells secrete numerous cytokines with different immunoregulatory and inflammatory properties, but induced CD71+ erythroid cells differ in the production of immunoregulatory molecules from natural adult bone marrow CD71+ erythroid cells. The overall cytokine profiles are similar between the two types of CD71+ erythroid cells, but the production of interleukin, chemokines, and growth factors differs significantly between the two populations. The most significant differences between the two types of erythroblasts are presented for IL-1beta, IL-13, RANTES and IL-12 (p40): their secretion by natural bone marrow CD71+ erythroid cells is more than 10 times higher than the secretion by induced CD71+ erythroid cells.

Most of the cytokines in the culture supernatant of CD71+ erythroid cells can be attributed to mediators that provide chemotaxis and migration of resting memory cells, activated CD4+ and CD8+ T cells, natural killer and dendritic cells, growth of lymphoid cells, progenitor T cells and mature T cells, as well as factors contributing to both the activation of T cells and the suppression of inflammatory reactions [37, 38]. T-cells are present in the bone marrow and make up approximately 3–8% of the total number of nucleated cells, and these T-cells usually show a lower CD4/CD8 ratio compared to blood T cells [39]. The bone marrow is a “reservoir” of memory T-cells as it contains central memory T-cells and effector memory T-cells [40, 41]. Most T-lymphocytes are circulating cells, and their localization in the bone marrow allows them to remain active [42]. In addition, T cells regulate normal physiological processes in the bone marrow, such as normal hematopoiesis and bone tissue homeostasis [43, 44]. Thus, it can be assumed that bone marrow CD71+ erythroid cells actively produce a spectrum of cytokines necessary for the functioning and survival of T-cells. According to our data, induced CD71+ erythroid produce less of these cytokines, possibly due to the lack of appropriate hematopoietic niches and microenvironment. On the other hand, induced CD71+ erythroid cells actively produce such cytokines as SCF, which promotes the proliferation of myeloid and lymphoid hematopoietic precursor cells in bone marrow cultures [45], as well as IL-3 and IL-5, which stimulate the formation of colonies of megakaryocytes, neutrophils, and macrophages from bone marrow cultures and growth of immature hematopoietic progenitors of BFU-E [46]. CD71+ erythroid cells secrete large amounts of G-CSF, which stimulates hematopoietic progenitor cells and promotes the formation and mobilization of bone marrow neutrophils, as well as the survival and chemotaxis of mature neutrophils [47]. Based on these data, it can be assumed that CD71+ erythroid cells, with the help of soluble factors, are capable of autocrine regulation not only of erythropoiesis, but also of the activity of other hematopoietic cells. Cytokines help create a microenvironment that promotes both self-renewal and recruitment of various cell populations, while the observed suppressive properties of CD71+ erythroid cells induced by us are mainly due to the observed low production of factors that stimulate the pro-inflammatory immune response [48, 49].

## Conclusion

Thus, differentiation of CD34+ cells in vitro induces an CD71+ erythroid cells phenotype with immunoregulatory activity different from that of natural bone marrow CD71+ erythroid cells. This view is supported by the agreement between our multiplex analysis and mRNA profiling results regarding differences in properties between these two groups of cells. Our findings are relevant and important, since CD71+ erythroid cells can have both a positive regulatory effect (in inflammatory and autoimmune diseases, pregnancy, and the neonatal period) and a negative regulatory effect (in cancer, infectious diseases, and anemia) on the mechanisms of the immune system [15]. Differentiation of erythrocytes in vitro is an important clinically significant process for modeling the study of the development of erythrocytes in normal and pathological hematopoiesis, for the development of therapeutic protocols aimed at the treatment of diseases associated with erythrocytes, such as sickle cell anemia, thalassemia, various anemias [1, 50, 51]. In addition to immunoregulatory properties, *in vitro* erythropoiesis is a good model for studying translational control in cell differentiation and physiology [52], antibody screening tools, disease models or developmental research tools [53]. Thus, when cultivating erythroblasts for clinical experimental studies, it is necessary to take into account their pronounced immunoregulatory activity.

## References

[1] Zivot A, Lipton JM, Narla A, Blanc L. Erythropoiesis: insights into pathophysiology and treatments in 2017. Mol Med. 2018, 24(1), 11. doi: 10.1186/s10020-018-0011-z 30134792 PMC6016880

[2] Koury S, Yarlagadda S, Moskalik-Liermo K, Popli N, Kim N, Apolito C, et al. Differential gene expression during terminal erythroid differentiation Genomics. 2007, 90(5), 574–82 doi: 10.1016/j.ygeno.2007.06.010 17764892 PMC2205530

[3] Huang P, Zhao Y, Zhong J, Zhang X, Liu Q, Qiu X, et al. Putative regulators for the continuum of erythroid differentiation revealed by single-cell transcriptome of human BM and UCB cells. Proc Natl Acad Sci U S A. 2020,117(23), 12868–12876. doi: 10.1073/pnas.1915085117 32457162 PMC7293633

[4] Morera D, MacKenzie SA. Is there a direct role for erythrocytes in the immune response? Vet Res. 2011, 42(1), 89. doi: 10.1186/1297-9716-42-89 21801407 PMC3199785

[5] Mohseni YR, Tung SL, Dudreuilh C, Lechler RI, Fruhwirth GO, Lombardi G. The Future of Regulatory T Cell Therapy: Promises and Challenges of Implementing CAR Technology. Front Immunol. 2020, 11, 1608. doi: 10.3389/fimmu.2020.01608 32793236 PMC7393941

[6] Law AMK, Valdes-Mora F, Gallego-Ortega D. Myeloid-Derived Suppressor Cells as a Therapeutic Target for Cancer. Cells. 2020, 9(3), 561. doi: 10.3390/cells9030561 32121014 PMC7140518

[7] Hmadcha A, Martin-Montalvo A, Gauthier BR, Soria B, Capilla-Gonzalez V. Therapeutic Potential of Mesenchymal Stem Cells for Cancer Therapy. Front Bioeng Biotechnol. 2020, 8, 43. doi: 10.3389/fbioe.2020.00043 32117924 PMC7013101

[8] Shahbaz S, Bozorgmehr N, Koleva P, Namdar A, Jovel J, Fava RA, et al. CD71+VISTA+ erythroid cells promote the development and function of regulatory T cells through TGF-β. PLoS Biol. 2018, 16(12), e2006649. doi: 10.1371/journal.pbio.2006649 30550561 PMC6310287

[9] Dunsmore G, Bozorgmehr N, Delyea C, Koleva P, Namdar A, Elahi S. Erythroid Suppressor Cells Compromise Neonatal Immune Response against Bordetella pertussis. J Immunol., 2017,. 199, 2081–2095. doi: 10.4049/jimmunol.1700742 28779022

[10] Elahi S, Ertelt JM, Kinder JM, Jiang TT, Zhang X, Xin L, et al. Immunosuppressive CD71+ erythroid cells compromise neonatal host defence against infection. Nature, 2013, 504, 158–62. doi: 10.1038/nature12675 24196717 PMC3979598

[11] Delyea C, Bozorgmehr N, Koleva P, Dunsmore G, Shahbaz S, Huang V, et al. CD71+ Erythroid Suppressor Cells Promote Fetomaternal Tolerance through Arginase-2 and PDL-1. J Immunol., 2018, 200(12), 4044–4058. doi: 10.4049/jimmunol.1800113 29735482

[12] Grzywa TM, Justyniarska M, Nowis D, Golab J. Tumor Immune Evasion Induced by Dysregulation of Erythroid Progenitor Cells Development. Cancers (Basel). 2021, 13(4), 870. doi: 10.3390/cancers13040870 33669537 PMC7922079

[13] Huerga Encabo H, Grey W, Garcia-Albornoz M, Wood H, Ulferts R, Aramburu IV, et al. Human Erythroid Progenitors Are Directly Infected by SARS-CoV-2: Implications for Emerging Erythropoiesis in Severe COVID-19 Patients. Stem Cell Reports. 2021, 16(3), 428–436. doi: 10.1016/j.stemcr.2021.02.001 33581053 PMC7862909

[14] Kronstein-Wiedemann R, Stadtmüller M, Traikov S, Georgi M, Teichert M, Yosef H, et al. SARS-CoV-2 Infects Red Blood Cell Progenitors and Dysregulates Hemoglobin and Iron Metabolism. Stem Cell Rev Rep. 2022, 18(5), 1809–1821. doi: 10.1007/s12015-021-10322-8 35181867 PMC8856880

[15] Grzywa TM, Nowis D, Golab J. The role of CD71+ erythroid cells in the regulation of the immune response. Pharmacol Ther. 2021, 228, 107927. doi: 10.1016/j.pharmthera.2021.107927 34171326

[16] Xu C, He J, Wang H, Zhang Y, Wu J, Zhao L, et al. Single-cell transcriptomic analysis identifies an immune-prone population in erythroid precursors during human ontogenesis. Nat Immunol. 2022, 23(7), 1109–1120. doi: 10.1038/s41590-022-01245-8 35761081

[17] Simamura E, Arikawa T, Ikeda T, Shimada H, Shoji H, Masuta H, et al. Melanocortins contribute to sequential differentiation and enucleation of human erythroblasts via melanocortin receptors 1, 2 and 5. PLoS One. 2015, 10(4), e0123232. doi: 10.1371/journal.pone.0123232 25860801 PMC4393082

[18] Chen K, Liu J, Heck S, Chasis JA, An X, Mohandas N. Resolving the distinct stages in erythroid differentiation based on dynamic changes in membrane protein expression during erythropoiesis. Proc Natl Acad Sci U S A. 2009, 106(41), 17413–8. doi: 10.1073/pnas.0909296106 19805084 PMC2762680

[19] Aimaitijiang A, Tabu K, Wang W, Nobuhisa I, Taga T. Glioma cells remotely promote erythropoiesis as a self-expanding strategy of cancer stem cells. Genes Cells. 2022, 27(1), 25–42. doi: 10.1111/gtc.12908 34837452

[20] Kassem O, Al-Saleh A, Azizieh F, Dingle K. CytokineExplore: An Online Tool for Statistical Analysis of Cytokine Concentration Datasets. J Inflamm Res. 2020, 13, 401–410. doi: 10.2147/JIR.S253255 32801833 PMC7406373

[21] Mehrotra A, Leventhal J, Purroy C, Cravedi P. Monitoring T cell alloreactivity. Transplant Rev (Orlando). 2015, 29(2), 53–9. doi: 10.1016/j.trre.2014.11.001 25475045 PMC6697151

[22] Neildez-Nguyen TM, Wajcman H, Marden MC, Bensidhoum M, Moncollin V, Giarratana MC, et al. Human erythroid cells produced ex vivo at large scale differentiate into red blood cells in vivo. Nat Biotechnol. 2002, 20(5), 467–72. doi: 10.1038/nbt0502-467 11981559

[23] Chen J, Qiao YD, Li X, Xu JL, Ye QJ, et al. Intratumoral CD45+CD71+ erythroid cells induce immune tolerance and predict tumor recurrence in hepatocellular carcinoma. Cancer Lett. 2021, Feb 28;499:85–98. doi: 10.1016/j.canlet.2020.12.003 33279623

[24] Zhao L, He R, Long H, Guo B, Jia Q, Qin D, et al. Late-stage tumors induce anemia and immunosuppressive extramedullary erythroid progenitor cells. Nat Med. 2018, 24(10), 1536–1544. doi: 10.1038/s41591-018-0205-5 30297899 PMC6211844

[25] Shahbaz S, Xu L, Osman M, Sligl W, Shields J, Joyce M, et al. Erythroid precursors and progenitors suppress adaptive immunity and get invaded by SARS-CoV-2. Stem Cell Reports. 2021 May 11;16(5):1165–1181. doi: 10.1016/j.stemcr.2021.04.001 ; PMCID: PMC8111797 33979601 PMC8111797

[26] Saito S, Shahbaz S, Sligl W, Osman M, Tyrrell DL, Elahi S. Differential Impact of SARS-CoV-2 Isolates, Namely, the Wuhan Strain, Delta, and Omicron Variants on Erythropoiesis. Microbiol Spectr. 2022 Aug 31;10(4):e0173022. doi: 10.1128/spectrum.01730-22 Epub 2022 Aug 9. ; PMCID: PMC9430111 35943266 PMC9430111

[27] Grzywa TM, Sosnowska A, Rydzynska Z, Lazniewski M, Plewczynski D, Klicka K, et al. Potent but transient immunosuppression of T-cells is a general feature of CD71+ erythroid cells. Commun Biol. 2021, 4(1), 1384. doi: 10.1038/s42003-021-02914-4 34893694 PMC8664950

[28] Hu J, Liu J, Xue F, Halverson G, Reid M, Guo A, et al. Isolation and functional characterization of human erythroblasts at distinct stages: implications for understanding of normal and disordered erythropoiesis in vivo. Blood. 2013, 121(16), 3246–53. doi: 10.1182/blood-2013-01-476390 Epub 2013 Feb 19. ; PMCID: PMC3630836. 23422750 PMC3630836

[29] Paulson RF, Ruan B, Hao S, Chen Y. Stress Erythropoiesis is a Key Inflammatory Response. Cells. 2020, 9(3), 634. doi: 10.3390/cells9030634 32155728 PMC7140438

[30] Sennikov SV, Eremina LV, Samarin DM, Avdeev IV, Kozlov VA. Cytokine gene expression in erythroid cells. Eur Cytokine Netw. 1996, 7(4), 771–4. 9010680

[31] Sennikov SV, Inzhelevskaya TV, Eremina LV, Kozlov VA. Regulation of functional activity of bone marrow hemopoietic stem cells by erythroid cells in mice. Bull Exp Biol Med. 2000, 130(12), 1159–61. 11276310

[32] Sennikov SV, Krysov SV, Injelevskaya TV, Silkov AN, Kozlov VA. Production of cytokines by immature erythroid cells derived from human embryonic liver. Eur Cytokine Netw. 2001, 12(2), 274–9. 11399516

[33] Sennikov SV, Injelevskaya TV, Krysov SV, Silkov AN, Kovinev IB, Dyachkova NJ, et al. Production of hemo- and immunoregulatory cytokines by erythroblast antigen+ and glycophorin A+ cells from human bone marrow. BMC Cell Biol. 2004, 5(1), 39. doi: 10.1186/1471-2121-5-39 15488155 PMC524510

[34] Denisova VV, Kulagin AD, Lisukov IA, Kryuchkova IV, Sizikova SA, Sennikov SV, et al. Cytokine-producing activity of bone marrow erythrokaryocytes and its regulation under normal conditions. Bull Exp Biol Med. 2007, 143(2), 218–21. doi: 10.1007/s10517-007-0055-5 17970206

[35] Perik-Zavodskii R, Perik-Zavodskaya O, Shevchenko Y, Denisova V, Nazarov K, Obleuhova I, et al. Immune Transcriptome and Secretome Differ between Human CD71+ Erythroid Cells from Adult Bone Marrow and Fetal Liver Parenchyma. Genes (Basel). 2022, 13(8), 1333. doi: 10.3390/genes13081333 35893070 PMC9330402

[36] Sennikov SV, Krysov SV, Silkov AN, Injelevskaya TV, Kozlov VA. Production of IL-10, TNF-alpha, IFN-gamma, TGF-beta1 by different populations of erythroid cells derived from human embryonal liver. Cytokine 2002, 17(4), 221–5. doi: 10.1006/cyto.2001.0975 11991675

[37] Lissoni P., Messina G., Pelizzoni F., Rovelli F., Brivio F., Monzon A., et al. The Fascination of Cytokine Immunological Science. J. Infect. 2020, 3, 18–28. doi: 10.29245/2689-9981/2020/1.1155;

[38] Hughes CE, Nibbs RJB. A guide to chemokines and their receptors. FEBS J. 2018, 285(16), 2944–2971. doi: 10.1111/febs.14466 Epub 2018 Apr 24. 29637711 PMC6120486

[39] Di Rosa F, Pabst R. The bone marrow: a nest for migratory memory T cells. Trends Immunol. 2005, 26(7), 360–6. doi: 10.1016/j.it.2005.04.011 15978522

[40] Mazo IB, Honczarenko M, Leung H, Cavanagh LL, Bonasio R, Weninger W, et al. Bone marrow is a major reservoir and site of recruitment for central memory CD8+ T cells. Immunity 2005, 22(2), 259–70. doi: 10.1016/j.immuni.2005.01.008 15723813

[41] Zhang X, Dong H, Lin W, Voss S, Hinkley L, Westergren M, et al. Human bone marrow: a reservoir for "enhanced effector memory" CD8+ T cells with potent recall function. J Immunol. 2006, 177(10), 6730–7. doi: 10.4049/jimmunol.177.10.6730 17082586

[42] Bonomo A, Monteiro AC, Gonçalves-Silva T, Cordeiro-Spinetti E, Galvani RG, Balduino A. A T Cell View of the Bone Marrow. Front Immunol. 2016, May 17;7:184. doi: 10.3389/fimmu.2016.00184 27242791 PMC4868947

[43] Monteiro JP, Benjamin A, Costa ES, Barcinski MA, Bonomo A. Normal hematopoiesis is maintained by activated bone marrow CD4+ T cells. Blood. 2005, 105(4), 1484–91. doi: 10.1182/blood-2004-07-2856 15514013

[44] Li Y, Toraldo G, Li A, Yang X, Zhang H, Qian WP, et al. B cells and T cells are critical for the preservation of bone homeostasis and attainment of peak bone mass in vivo. Blood. 2007, 109(9), 3839–48. doi: 10.1182/blood-2006-07-037994 17202317 PMC1874582

[45] Li Z, MacDougald OA. Stem cell factor: the bridge between bone marrow adipocytes and hematopoietic cells. Haematologica. 2019, 104(9), 1689–1691. doi: 10.3324/haematol.2019.224188 31473604 PMC6717568

[46] Dougan M, Dranoff G, Dougan SK. GM-CSF, IL-3, and IL-5 Family of Cytokines: Regulators of Inflammation. Immunity. 2019, 50(4), 796–811. doi: 10.1016/j.immuni.2019.03.022 30995500 PMC12512237

[47] Cetean S, Căinap C, Constantin AM, Căinap S, Gherman A, Oprean L, et al. The importance of the granulocyte-colony stimulating factor in oncology. Clujul Med. 2015, 88(4), 468–72. doi: 10.15386/cjmed-531 26732055 PMC4689238

[48] Hershey GK. IL-13 receptors and signaling pathways: an evolving web. J Allergy Clin Immunol. 2003, 111(4), 677–90; quiz 691. doi: 10.1067/mai.2003.1333 12704343

[49] Iwaszko M, Biały S, Bogunia-Kubik K. Significance of Interleukin (IL)-4 and IL-13 in Inflammatory Arthritis. Cells 2021, 10(11), 3000. doi: 10.3390/cells10113000 34831223 PMC8616130

[50] Cannon M, Phillips H, Smith S, Mitchell S, Landes K, Desai P, et al. Red blood cells differentiated in vitro using sequential liquid and semi-solid culture as a pre-clinical model. Exp Hematol Oncol. 2021, 10(1), 50. doi: 10.1186/s40164-021-00244-z 34715921 PMC8555309

[51] Fibach E. Erythropoiesis In Vitro-A Research and Therapeutic Tool in Thalassemia. J Clin Med. 2019, 8(12), 2124. doi: 10.3390/jcm8122124 31810354 PMC6947291

[52] Vatikioti A, Karkoulia E, Ioannou M, Strouboulis J. Translational regulation and deregulation in erythropoiesis. Exp Hematol. 2019, 75, 11–20. doi: 10.1016/j.exphem.2019.05.004 31154069

[53] Bernecker C, Ackermann M, Lachmann N, Rohrhofer L, Zaehres H, Araúzo-Bravo MJ, et al. Enhanced Ex Vivo Generation of Erythroid Cells from Human Induced Pluripotent Stem Cells in a Simplified Cell Culture System with Low Cytokine Support. Stem Cell Dev. 2019, 28(23), 1540–1551. doi: 10.1089/scd.2019.0132 31595840 PMC6882453

